# Dyspepsia—Its Causes and Treatment

**Published:** 1880-02

**Authors:** R. W. Westmoreland

**Affiliations:** Atlanta, Ga.


### DYSPEPSIA—ITS CAUSES AND TREATMENT.

By R. W. WESTMORELAND, M.D., Atlanta, Ga.

In the treatment of diseases, the first point of importance is to consider the causes and effects that act in producing the individual malady. Too often is it the case that the resort to a routine system of prescribing robs the practitioner of much satisfaction and professional eclat, while the patient derives no benefit whatever from the "classic" plan of treatment instituted. In dyspepsia particularly does this state of affairs often obtain. Almost totally ignoring the fact that the causes of the disease are nearly as various as the individuals affected, many practitioners act on the idea that gentian, bismuth, mercury, and so on through the catalogue of gastric and hepatic remedies, are in every instance to be given, regardless of any special indication. The actual pathology of the affection makes manifest the inefficiency of any such indiscriminate practice. Take, for instance, a patient suffering from digestive derangement as the result of some reflex nervous disturbance where, at the point whence the nerves supplying the digestive apparatus are given off, the spinal function is perturbed. Manifestly in this case the exhibition of remedies to a deranged stomach would have but temporary effect, if any at all, and the patient be more apt to grow worse than better.

The more rational therapeutical indication would direct to give remedies that would overcome the spinal derangement by removing the cause of the original reflex disturbance.

As we have dyspeptic difficulties arising from primary liver, stomach and pancreatic derangement, as well as reflex spinal and intestinal disturbance, the prime rational course to pursue would be a direct differentiation as to these operating causes, when the therapeusis would be made clearer.

Each individual suffering with dyspepsia is a law unto himself. No doubt disposition or temperament has much to do in characterizing each individual disease, and in predisposing to peculiar complications. The so-called bilious temperament will present most frequently that form in which a perturbed liver will be the fundamental cause. In these cases the intimate sympathy existing between the stomach and liver will always lead to disease of the former viscus as a complication of the original difficulty. Obstinate constipation is a common concomitant of this form of the malady, and the train of symptoms presented would seem to indicate a corresponding complication in therapeutics. Such, however, we have not found to be the case, and generally find that a mercurial cathartic, with restricted diet subsequently, is sufficient for most cases.

Again we find as the source of the dyspeptic ailment an insufficiency of pancreatic function. In this instance the history of the case discloses the fact that a fatty diet disagrees with the patient; the bowels are constipated and the appetite varying. Pancreatine, by supplying the emulsifying principle, with aloes to stir up the torpid bowels, will usually suffice to effect a cure. Restricted and suitable diet will always be found necessary, and should be strictly enforced, for it is often the case that a departure from temperate eating is the primary cause of the difficulty.

In those cases of obstinate constipation, where from a sluggish acting liver the peristaltic motion of the bowels is modified, belladonna, in connection with rhubarb, has been found to act very beneficially. In fact, there are those who rely solely on small doses, often repeated, of belladonna, without any other agent, and report good results.

Mechanical irritants, such as pulverized charcoal, sand and so forth, have been suggested and used beneficially in constipation dependent upon spinal innervation, but as their benefit is but temporary, they should be given in connection with nux vomica or other remedies calculated to have the effect of stimulating the weakened spinal function. Could this diversity of the causes of dyspepsia be appreciated generally, there is no doubt but that the unsatisfactory results usually attending the treatment of the disease would be lessened, and the satisfaction and relief of the many suffering therefrom be greatly increased. In scarcely any other affection is the discrimination of causes and the recognition of direct results so essential as in dyspepsia. In no other is the foolish abuse of routine practice so blindly and so generally followed. A primarily simple disease is too often aggravated to become a hydra whose protean complications detrimentally influence every physiological function.

